# Giant Retinal Astrocytoma: A Case Report of an Uncommon Presentation of Tuberous Sclerosis in a Young Female

**DOI:** 10.1155/2024/5559615

**Published:** 2024-04-24

**Authors:** Keval Thakkar, Fnu Raveena, Aakash Kumar, Doongro Mal, Dileep Kumar, Neha Ahuja, Rahul Mandhan, Aqsa Baig, Manjeet Singh, Heeya Shah, Taha Sajjad, Mansi Singh

**Affiliations:** ^1^Georgetown University, District of Columbia, USA; ^2^Ghulam Muhammad Mahar Medical College, Sukkur University, Larkana, Pakistan; ^3^Liaquat National Medical College, Karachi, Pakistan; ^4^Dow University of Health Sciences, Karachi, Pakistan; ^5^Liaquat University of Medical & Health Sciences, Karachi, Pakistan; ^6^Chandka Medical College, Larkana, Pakistan; ^7^Jinnah Sindh Medical University, Karachi, Pakistan; ^8^OSF Saint Francis Medical Centre, Peoria, IL, USA; ^9^University of South Carolina, Lancaster, PA, USA; ^10^Mountain Vista Medical Center, Phoenix, AZ, USA; ^11^Bogomolets National Medical University, Kyiv, Ukraine

## Abstract

Tuberous sclerosis (TS) is a rare multisystem autosomal dominant genetic disorder with characteristic pathognomonic genetic mutations involving the TSC (tuberous sclerosis complex) group of genes. Ocular signs are fairly common and include an achromic patch and retinal astrocytic hamartomas, which usually have a maximum size of between 0.5 and 5 mm. The incidence of tuberous sclerosis is estimated to be 1 in 5000−10,000 individuals, with both familial and sporadic cases reported. The diagnostic criteria for tuberous sclerosis include the presence of major and/or minor clinical features as well as genetic mutations. We present the case of a 15-year-old girl, presented with a history of seizures and blurred vision. Physical examination revealed angiofibroma on the face. Further evaluation, including contrast-enhanced MRI of the brain and ophthalmological consultation, led to the diagnosis of tuberous sclerosis. Additional imaging studies confirmed the presence of subependymal giant cell astrocytoma, retinal astrocytoma, lymphangioleiomyomatosis in the lungs, and renal angiomyolipoma. This case highlights the importance of considering tuberous sclerosis in patients presenting with seizures and ocular symptoms. This case sheds light on early diagnosis and appropriate management which are crucial in preventing complications and improving patient outcomes.

## 1. Introduction

Tuberous sclerosis (TS), also known as the eponymous Bourneville-Pringle's disease, is a rare genetic disorder inherited in an autosomal dominant pattern, affecting multiple systems, including the skin, brain, viscera, and eyes leading to the development of hamartomatous tumors. This disorder exhibits variable phenotypic expression [1]. The incidence of TS in the general population averages 1 in 5000−10,000 individuals, with no gender or racial predilection. Approximately, one-third of cases have a positive family history, while the remaining are sporadic [2].

As per the 2021 International Tuberous Sclerosis Complex Consensus Conference recommendations, a definitive diagnosis of TS can be made if the condition presents with two major features, one major feature along with two or more minor features, or a pathognomonic genetic mutation. Alternatively, a diagnosis can be established if it exhibits one major feature or two or more minor features. In addition, the detection of a pathogenic variant in either TSC1 or TSC2 is adequate for diagnosing or predicting tuberous sclerosis complex (TSC), irrespective of the clinical manifestations. These diagnostic criteria are summarized in Table 1. It is essential to note that the presence of lymphangioleiomyomatosis (LAM) and angiomyolipomas together, without other features, does not meet definitive diagnostic criteria for TS [1, 3].

Ocular symptoms in TSC can manifest in various ways affecting the retina, optic nerve, adnexa, or, less commonly, the choroid. However, for diagnostic purposes, only retinal symptoms such as retinal astrocytic hamartomas and achromic patches are considered. Even though they are most often seen in people with tuberous sclerosis complex (TSC), retinal astrocytic hamartomas are not unique to TSC. They are rarely seen in people with neurofibromatosis or even in healthy people. The distribution is usually multifocal or bilateral in TSC, unlike solitary lesions seen in other conditions [1]. Retinal astrocytomas are glial tumors of the retinal nerve fibre layer, morphologically classified into three distinct types [4]. A review of the prior published literature reveals that most lesions range between 0.5 and 5 mm in diameter, and larger lesions are considered exceptional [5].

We present the uncommon presentation of tuberous sclerosis in a young female patient. The patient presented with a history of seizure episodes and blurred vision, which led to further diagnostic workup and the identification of tuberous sclerosis-related manifestations in the brain, eyes, lungs, and kidneys.

## 2. Case Presentation

We present the case of a 15-year-old female who presented to the emergency department due to seizure episodes that had been occurring on and off for the past 3 months. These episodes were characterized by tonic-clonic limb movements followed by a loss of consciousness, which is a hallmark of seizure activity. The patient also complained about blurred vision. Although no significant past medical history and family history were noted.

On physical examination, the patient's vitals showed a blood pressure of 130/80 mmHg, a heart rate of 76/min, respiratory rate of 15/min, temperature of 37.3°C, and SpO_2_ of 96%. The patient had maculopapular hyperpigmented skin lesions over her face. The rest of the systematic examination was unremarkable.

**Figure 1:**
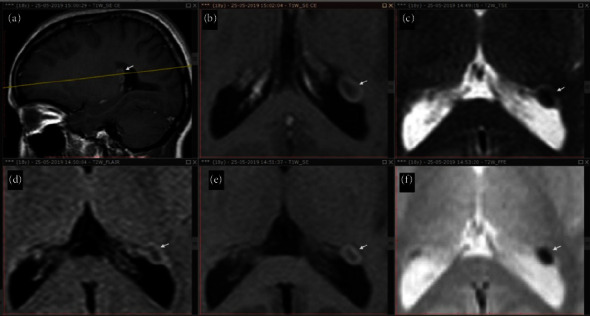
A contrast-enhanced MRI of the brain shows: (a) FLAIR; lesion: peripheral hyperintensity and central hypointensity. (b) T1W: proximity to the foramen of Monroe; lesion: peripheral hyperintensity and central hypointensity. (c) T2; lesion: peripheral hyperintensity and central isointensity. Peripherally hyperintense with the central hypointensity on FLAIR (d), peripherally hyperintense with the central isointensity on T1W (e), and blooming on T2 (f).

After proper informed consent was obtained, on further evaluation of the contrast-enhanced MRI of the brain, a distinctive round-to-oval-enhancing mass lesion was prominently observed. This lesion originates from the antrum of the left lateral ventricle, positioned in close proximity to the foramen of Monroe. Notably, it exhibits a peripheral hyperintense pattern with central hypointensity on the FLAIR sequence. Moreover, the lesion presents a peripherally hyperintense configuration with central isointensity on the T1-weighted image and exhibits blooming indicating tubers with hyperintense lesions on the T2 sequence, as shown in Figure 1. The lesion was consistent morphologically with a subependymal giant cell astrocytoma in close proximity to the left foramen of Monroe.

**Figure 2:**
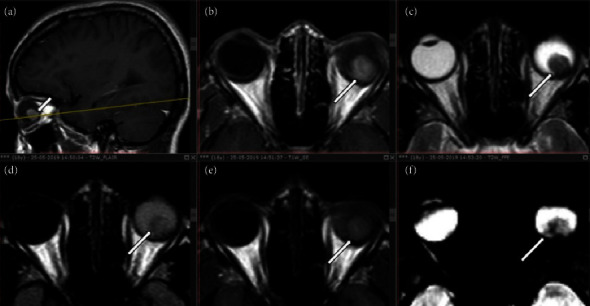
Contrast-enhanced magnetic resonance imaging (MRI) of the brain shows a round to oval enhancing mass lesion (white arrows) arising from the juxtapapillary location of the left globe (a and b); appearing heterogeneously hypointense compared to surrounding media on the T2W image (c). Iso to hypointense on FLAIR (d), mildly hyperintense compared to surrounding media on T1W (e), and blooming foci on T2 (f).

A solitary ocular lesion in the posterior aspect of the left globe measures approximately 1.2 × 1.3 × 1.4 cm. This lesion appears heterogeneously hypointense compared to the surrounding media, as shown in Figure 2.

The ophthalmologic consultation resulted in a diagnosis of retinal astrocytoma, although the assessment was significantly hindered by hazy media. During the conventional slit-lamp examination, only secondary postoperative changes were observable, providing limited diagnostic information.

After the initial consultation with an ophthalmologist, the patient had a full diagnostic workup. This included a second ophthalmologic exam, contrast-enhanced computed tomography (CECT) scans of the brain, orbit, thorax, and abdomen, and an ultrasonography (USG) of the abdomen. The CECT brain and orbital imaging confirmed the earlier findings observed on the contrast-enhanced magnetic resonance imaging (CEMRI) brain, reinforcing the presence of the identified lesions.

**Figure 3:**
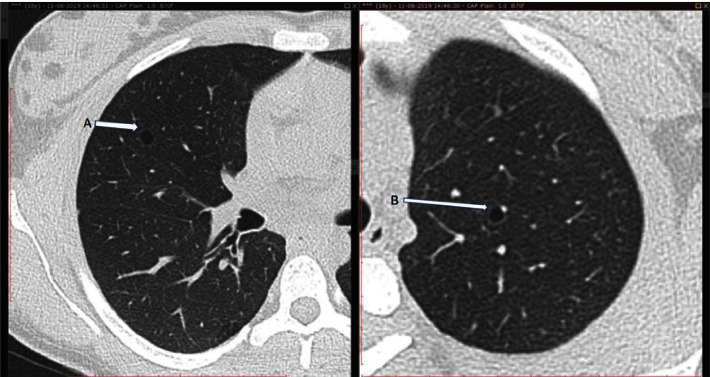
CT thorax shows round, thin-walled cystic lesions in the lateral segment of the right middle lobe (A) and the apical-posterior segment of the left upper lobe (B).

Intriguingly, during the CECT thorax assessment, two lung lesions consistent with lymphangioleiomyomatosis (LAM) were identified, as shown in Figure 3, raising concerns about potential systemic involvement.

**Figure 4:**
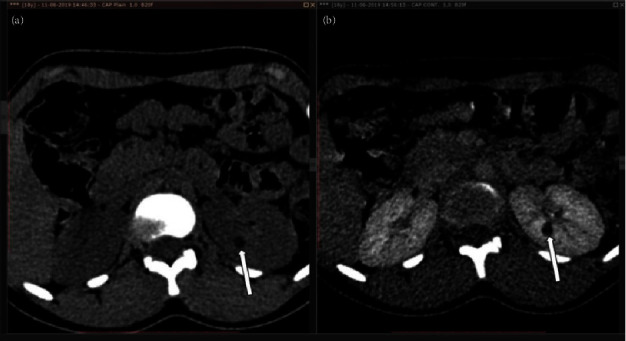
Plain (a) and contrast (b) CT abdomen shows a hypo attenuating (average-12 HU on plain CT) mass lesion in the interpolar cortex of the left kidney, with evidence of mild peripheral enhancement.

**Figure 5:**
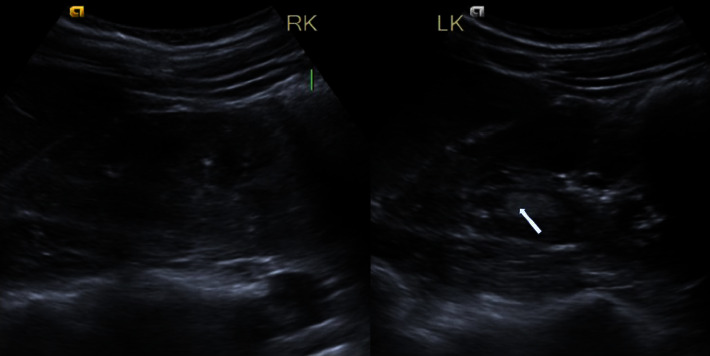
Ultrasonography (USG) of the left kidney: solitary hyperechoic angiomyolipoma (white arrow) in interpolar cortex and bilateral multiple small (2-3 mm) echogenic foci and suspected smaller angiomyolipomas.

In addition, the CECT abdomen revealed a solitary lesion consistent with a left renal angiomyolipoma, as shown in Figure 4. Furthermore, the USG KUB examination disclosed a solitary hyperechoic round to oval lesion in the interpolar cortex of the left kidney, approximately measuring 12 × 9 mm, displaying internal vascularity, which is highly suggestive of an angiomyolipoma. Moreover, multiple small (2–3 mm) round to oval variable-sized echogenic foci were observed in the bilateral renal cortices, likely representing smaller angiomyolipomas, as shown in Figure 5.

The patient was given treatment for seizures with antiseizure medications, advised with lifestyle modification, and sun protection measures, and recommended for follow-up checkups.

Considering the cumulative findings of the cerebral, pulmonary, and renal manifestations, this clinical presentation strongly suggests a diagnosis of tuberous sclerosis, a rare genetic disorder characterized by the development of benign tumors in various organs, including the brain, kidneys, lungs, and skin. Further genetic testing and clinical evaluation may be necessary to confirm this diagnosis and guide appropriate management. Following confirmation, the patient has been kept under observation with regular follow-up.

## 3. Discussion

Tuberous sclerosis complex (TSC) is an autosomal dominant genetic disorder with an incidence of 1 in 5000–10,000 live births, primarily caused by pathogenic variants in either the TSC1 or TSC2 genes. These mutations lead to overactivation of the mechanistic target of rapamycin (mTOR) pathway. The mTOR pathway is a critical cellular signaling pathway that plays a central role in various cellular processes, including cell growth, proliferation, metabolism, and autophagy. In the context of tuberous sclerosis complex (TSC), the mTOR pathway is dysregulated due to genetic mutations in the TSC1 or TSC2 genes.

Normal Regulation: In healthy cells, the mTOR pathway is tightly regulated. It acts as a cellular “sensing” mechanism that responds to various signals, including nutrient availability and growth factors.

TSC1 and TSC2 Genes: In TSC, there are mutations in either the TSC1 or TSC2 genes. These genes code for proteins known as hamartin (TSC1) and tuberin (TSC2), which normally work together to inhibit mTOR signaling. Due to mutations in TSC1 or TSC2, the hamartin-tuberin complex is disrupted. This leads to the dysregulation of the mTOR pathway. In the absence of a functional hamartin-tuberin complex, mTOR becomes hyperactive, leading to increased signaling along this pathway. Hyperactive mTOR promotes cell growth and division, and it inhibits the normal process of autophagy (the cellular recycling mechanism). This can result in the formation of benign tumors called hamartomas in various organs, including the brain, skin, kidneys, and heart. The dysregulated mTOR pathway is responsible for many of the clinical manifestations of TSC, including the development of hamartomas, seizures, cognitive impairments, and other symptoms associated with the condition. Because of the central role of the mTOR pathway in TSC, targeted therapies such as mTOR inhibitors (e.g., sirolimus and everolimus) have been developed and are used to treat some of the manifestations of the disease. These drugs aim to inhibit the overactive mTOR pathway and reduce the growth of tumors [6–9].

De novo pathogenic variants account for about 80% of TSC cases, with TSC2 variants being more common. Familial cases, on the other hand, have roughly equal proportions of TSC1 and TSC2 pathogenic variants. TSC exhibits high phenotypic variability in terms of age of onset, disease severity, and associated symptoms due to diverse genotypes, making its clinical presentation highly variable [10–12].

Tuberous sclerosis complex (TSC) is a complex disorder with a wide range of clinical manifestations. Seizures are one of the most common features of TSC and can present in various forms, including focal seizures, generalized seizures, and epileptic spasms [13]. In our case, the patient's initial presentation was a seizure, and owing to this, we did brain imaging. Brain imaging is essential for the diagnosis and management of TSC.

Ocular retinal astrocytic hamartomas are distinctive masses within the retinal nerve fibre layer, characterized by disorganized astrocytic proliferation and expression of glial fibrillary acid protein (GFAP) [14]. These hamartomas exhibit two histopathological patterns: one with an interlacing stroma of spindle-shaped cells and scattered polygonal cells, and another with large gemistocytic astrocytes featuring abundant glassy cytoplasm. Similar to other focal brain and skin manifestations, these lesions result from two-hit mutations involving the TSC1 or TSC2 genes. Working in conjunction with TBC1D7, these genes play a crucial role in regulating the mTOR pathway, serving as tumor suppressor genes. Retinal astrocytic hamartomas are usually linked to TSC, but they can also be caused by neurofibromatosis, retinitis pigmentosa, Best's disease, and Leber's congenital amaurosis [15–17]. Differentiating them from similar conditions, such as retinoblastoma, persistent hyperplastic primary vitreous, or choroidal melanoma, can be challenging. However, clinical and radiological criteria, along with the characteristic mulberry-like appearance and minimal retinal detachment, are crucial in the diagnosis [18].

Retinal astrocytic hamartomas in the TSC typically exhibit low malignant potential and remain relatively small, measuring between 0.5 and 5 mm in maximum dimension. They tend to remain stable over the course of the disease, presenting either unilaterally or bilaterally and in a solitary or multifocal distribution [5]. However, our patient's case deviates from this usual trend in two significant ways. First, the hamartoma is larger than the typical size, exceeding 1 cm in all dimensions. Second, it is associated with vitreous hemorrhage, which is a rare complication of astrocytomas. Despite these variations, the clinical and radiological diagnosis of TSC, coupled with the distinctive appearance of the hamartoma, the differentiation from retinoblastoma, which typically presents with gross retinal detachment. These unique characteristics emphasize the importance of considering diverse presentations of retinal complications in TSC and the significance of a thorough diagnostic evaluation.

Renal angiomyolipomas are another common feature of TSC and can be detected using imaging techniques such as ultrasound or computed tomography (CT). Renal angiomyolipomas can range from benign findings to life-threatening conditions. Treatment aims to preserve renal function and may include surgery or embolization. Advancements in understanding angiomyolipoma biology offer hope for future drug therapies [19]. Our case study presents a left renal angiomyolipoma confirmed on the CECT abdomen and further characterized through USG KUB. The main lesion measured 12 × 9 mm with internal vascularity, consistent with an angiomyolipoma. Given the potential multiorgan involvement, a multidisciplinary approach is essential for comprehensive patient care. This article aims to update ophthalmologists on molecular insights and future therapeutic possibilities for renal angiomyolipomas.

Management of TSC involves a multidisciplinary approach, with treatment aimed at controlling seizures, managing symptoms, and monitoring for complications. Antiepileptic medications are commonly used to control seizures, while other interventions such as surgical resection or embolization may be required for the management of other organ involvement. For all diagnosed patients, it is advisable to undergo a thorough dermatological assessment conducted by a seasoned specialist. The use of a Wood's lamp is beneficial in identifying hypomelanotic macules. Providing guidance on what to expect and potential treatments is recommended as part of anticipatory care. Sun protection is crucial for both adults and children due to the photosensitivity of hypomelanotic macules and the presence of mutations similar to those induced by ultraviolet radiation in angiofibroma. Intervention options include the use of mechanistic target of rapamycin inhibitors (mTORis), pulsed-dye or ablative lasers, or surgical excision, particularly for large, disfiguring lesions, or those prone to bleeding or causing discomfort [20].

## 4. Conclusion

Tuberous sclerosis (TS) is a rare genetic disorder that can present with a wide range of clinical manifestations. This case report highlights an unusual presentation of TS, with a young female patient presenting with a solitary atypical retinal astrocytic hamartoma of unusual dimensions with seizure. The diagnosis of TS was confirmed based on the presence of major and minor clinical features and the identification of genetic mutations. Management of TS involves a multidisciplinary approach, with treatment aimed at controlling symptoms and preventing complications. Further research is needed to better understand the pathogenesis and optimal management strategies for retinal astrocytic hamartomas in tuberous sclerosis.

## Figures and Tables

**Table 1:** Major and minor clinical, and genetic diagnostic criteria for definitive diagnosis of tuberous sclerosis.

Genetic diagnostic criteria	Major clinical diagnostic criteria	Minor clinical diagnostic criteria
(1) TSC 1 at 9q34.13 encoding hamartin protein (2) TSC 2 at 16p13.3 encoding tuberin protein	(1) Three or more hypomelanotic “ash-leaf” macules (more than 5 mm in diameter) (2) Three or more angiofibromas or fibrous cephalic plaques (3) Two or more ungual fibromas (4) Shagreen patch (5) Multiple retinal hamartomas (6) Cortical dysplasias (7) Subependymal nodules (8) Subependymal giant cell astrocytomas (9) Cardiac rhabdomyoma (10) Lymphangioleiomyomatosis (LAM) (11) Two or more angiomyolipomas	(1) Confetti skin lesions (2) More than three dental enamel pits (3) Two or more intraoral fibromas (4) Retinal achromic patch (5) Multiple renal cysts (6) Nonrenal hamartomas

## Data Availability

The datasets analyzed during the current study are available from the corresponding author upon reasonable request. In addition, comprehensive literature sources used for the literature review are cited appropriately within the manuscript.

## Additional Points

*Key Clinical Messages*. Giant cell astrocytoma, a manifestation of tuberous sclerosis complex (TSC), presents a rare genetic disorder characterized by a spectrum of clinical symptoms. These include seizures and the development of retinal astrocytic hamartomas. Recent molecular insights have unveiled potential avenues for targeted therapies, underscoring the significance of consistent follow-up appointments. This approach is crucial for adapting treatments over time and enhancing overall patient outcomes in cases of giant cell astrocytoma associated with TSC.
